# Mortality in Sickle Cell Anemia in Africa: A Prospective Cohort Study in Tanzania

**DOI:** 10.1371/journal.pone.0014699

**Published:** 2011-02-16

**Authors:** Julie Makani, Sharon E. Cox, Deogratius Soka, Albert N. Komba, Julie Oruo, Hadija Mwamtemi, Pius Magesa, Stella Rwezaula, Elineema Meda, Josephine Mgaya, Brett Lowe, David Muturi, David J. Roberts, Thomas N. Williams, Kisali Pallangyo, Jesse Kitundu, Gregory Fegan, Fenella J. Kirkham, Kevin Marsh, Charles R. Newton

**Affiliations:** 1 Department of Haematology and Blood Transfusion, Muhimbili University of Health and Allied Sciences, Dar-es-Salaam, Tanzania; 2 Nuffield Department of Medicine, University of Oxford, Oxford, United Kingdom; 3 MRC International Nutrition Group, London School of Hygiene & Tropical Medicine, London, United Kingdom; 4 Centre for Geographic Medicine Research Coast, Kenya Medical Research Institute (KEMRI), Kilifi, Kenya; 5 National Health Service Blood and Transplant–John Radcliffe Hospital, Oxford, United Kingdom; 6 INDEPTH Network of Demographic Surveillance Sites, Accra, Ghana; 7 Neurosciences Unit, University College London Institute of Child Health, London, United Kingdom; Stanford University School of Medicine, United States of America

## Abstract

**Background:**

The World Health Organization has declared Sickle Cell Anemia (SCA) a public health priority. There are 300,000 births/year, over 75% in Africa, with estimates suggesting that 6 million Africans will be living with SCA if average survival reaches half the African norm. Countries such as United States of America and United Kingdom have reduced SCA mortality from 3 to 0.13 per 100 person years of observation (PYO), with interventions such as newborn screening, prevention of infections and comprehensive care, but implementation of interventions in African countries has been hindered by lack of locally appropriate information. The objective of this study was to determine the incidence and factors associated with death from SCA in Dar-es-Salaam.

**Methods and Findings:**

A hospital-based cohort study was conducted, with prospective surveillance of 1,725 SCA patients recruited from 2004 to 2009, with 209 (12%) lost to follow up, while 86 died. The mortality rate was 1.9 (95%CI 1.5, 2.9) per 100 PYO, highest under 5-years old [7.3 (4.8–11.0)], adjusting for dates of birth and study enrollment. Independent risk factors, at enrollment to the cohort, predicting death were low hemoglobin (<5 g/dL) [3.8 (1.8–8.2); p = 0.001] and high total bilirubin (≥102 µmol/L) [1.7 (1.0–2.9); p = 0.044] as determined by logistic regression.

**Conclusions:**

Mortality in SCA in Africa is high, with the most vulnerable period being under 5-years old. This is most likely an underestimate, as this was a hospital cohort and may not have captured SCA individuals with severe disease who died in early childhood, those with mild disease who are undiagnosed or do not utilize services at health facilities. Prompt and effective treatment for anemia in SCA is recommended as it is likely to improve survival. Further research is required to determine the etiology, pathophysiology and the most appropriate strategies for management of anemia in SCA.

## Introduction

The greatest burden of sickle cell anemia (SCA) is in sub-Saharan Africa (SSA), where 75% of the 300,000 global births of affected children live[1], and estimates suggest that 50–80% of these patients will die before adulthood[2]. The World Health Organization estimate that 70% of SCA deaths in Africa are preventable with simple, cost-effective interventions such as early identification of SCA patients by newborn screening (NBS) and the subsequent provision of comprehensive care. Identification of risk factors has led to improved survival through targeted interventions. In the West, reported risk factors for death include infections, low hemoglobin and fetal Hb (HbF), high white blood cell count and hemolysis[3]–[5]. Comprehensive care includes prompt treatment of acute events and prophylaxis against infections, mainly with oral penicillin and vaccination against *Streptococcus pneumoniae.* Countries that have introduced these interventions have achieved significant reductions in mortality; with up to 94% surviving to 18 years in the United States of America (USA[6] and 99% to 20 years in the UK[7]. In most African countries, the lack of an evidence-base has led to inertia in terms of implementation of these interventions, such as penicillin prophylaxis[8]. One of the first steps in addressing this lack of knowledge is to provide an estimate of mortality rates to highlight the burden of disease due to SCA. The ideal approach is to establish a cohort of SCA patients, diagnosed at birth, and follow them up to determine rate and cause of death. However, most countries in Africa do not have NBS programs, therefore such evidence will rely on hospital-based studies. Information from such cohorts is biased, as it will on one hand consist of healthy survivors and on the other, will not identify those with mild disease who do not seek healthcare or those with severe disease who have died. This situation is similar to that in Jamaica and USA in the early 1970s, when NBS for SCA was not established and evidence relied on prospective studies in hospital-based cohorts, where most of the patients (92% and 65% respectively) were not identified at birth[4], [9]. Despite the limitations of hospital-based studies, these studies provided important evidence on morbidity and mortality due to SCA.

In 2004, we established prospective surveillance of SCA patients in a hospital in Tanzania. The frequency of the sickle heterozygous carrier state (AS) in Tanzania is 13% with an estimated annual births of 8,000 homozygous SS children, compared to 302 in Jamaica and 1,500 in the USA[10]. The overall aim of this study was to determine the burden of disease due to SCA in Tanzania by describing the spectrum of disease. Here, we report the rate and risk factors of mortality in Dar-es-Salaam, Tanzania.

## Methods

### Study area

The study was conducted at Muhimbili National Hospital (MNH), in Dar-es-Salaam, which is the administrative capital of Tanzania with a population of 4 million. The hospital is the national referral hospital but also serves as a first-level referral hospital for all SCA patients in Dar-es-Salaam. MNH provides health care to patients with SCA, where outpatient visits are scheduled at three monthly intervals for routine check-up. All SCA patients are prescribed folic acid (5 mg/day) and antimalarial prophylaxis (chloroquine) as recommended by the National Malaria Control Programme of Tanzania[11]. Penicillin prophylaxis is not part of standard care for SCA in Tanzania. There is no NBS in Tanzania to identify SCA individuals at birth. As part of the policy of Ministry of Health and Social Welfare of Tanzania, patients with SCA receive free health care. This study was integrated into the existing healthcare system with no attempts to recruit SCA patients or alter the system for referral and management.

Following institutional ethical approval (reference MU/RP/AEC/VOL XI/33), written informed consent, in the local language (Kiswahili), was obtained from parents or guardians of children and from patients who were above 18-years old.

### Procedures

Enrollment was done at outpatient clinic. At enrollment, a detailed history and examination was recorded onto standardized proformas. Blood samples were collected for a complete blood count (Pentra 60, Horiba ABX, Kyoto, Japan), hemoglobin electrophoresis (Helena, Sunderland, Tyne & Wear, UK), high performance liquid chromatography (Bio-Rad, Hercules, CA, USA) and biochemical analysis (Roche Cobas Mira, New York, USA or Abbott Architect, New York, USA). Nucleated RBC could not be differentiated from neutrophils by the haematology analyzer. All the SCA patients attending the clinic followed the existing clinical practice of referrals and diagnosis on the basis of clinical suspicion or family history. The management (diagnosis and treatment) followed the hospital guidelines, with no change in the normal clinical practice.

SCA individuals with acute illness were managed in the emergency medicine department or were hospitalized. During episodes of acute illness, patients were encouraged to contact their nearest health facility and follow the referral procedures through the public health care system. The decision to admit SCA patients was made by the attending clinician in the hospital casualty department, following criteria set by MNH**.**

SCA patients who did not attend clinic for more than 12 months were defined as defaulters and were actively contacted by telephone or, for those residents in Dar-es-Salaam, visited at home. Patients who were not found after three attempts of tracing were considered to be lost to follow-up.

### Statistical analysis

Data were analyzed using STATAv10 (StataCorp, College Station, TX, USA). The population prevalence of SCA was calculated from the enrolled SCA patients divided by the population of each ward as estimated in population census done in 2002. Wards are local government units, on which the census is based.

The study period started at enrollment clinic visit and the end was date of death or date last known to be alive. For patients who died outside the hospital where date of death could not be ascertained, we used the date of last hospital attendance. The overall and age-specific incident rate of mortality was estimated. This was calculated from the ratio of number of deaths divided by the number of person years of observation (PYO), expressed as mortality rates. The Cnaan and Ryan approach to data analysis was used[12], which takes into account patients entering and leaving the study cohort with the observation beginning after disease onset, which in this case was at birth. According to this method, a patient contributes to the population at risk for a given death only if that patient is enrolled in the study at the age at which death occurs. We modeled age at death rather than the length of time from enrollment to death to determine life expectancy, in an approach similar to that used by Platt *et al* [5]. The standardized mortality ratio was calculated from the age-specific mortality rate in Dar-es-Salaam (source: Tanzania national Bureau of statistics).

Data were summarized as means, medians or proportions. Logistic regression was used to analyze factors at enrollment associated with death, with results presented as odds ratios (ORs) with 95% confidence intervals (95%CIs). Multivariable logistic regression was used to identify independent associations with death, using variables that had significant association (p<0.05) on univariable analysis. For the SCA patients who died in hospital, the clinical and laboratory findings were reviewed and likely cause of death was ascertained.

## Results

**Figure 1:**
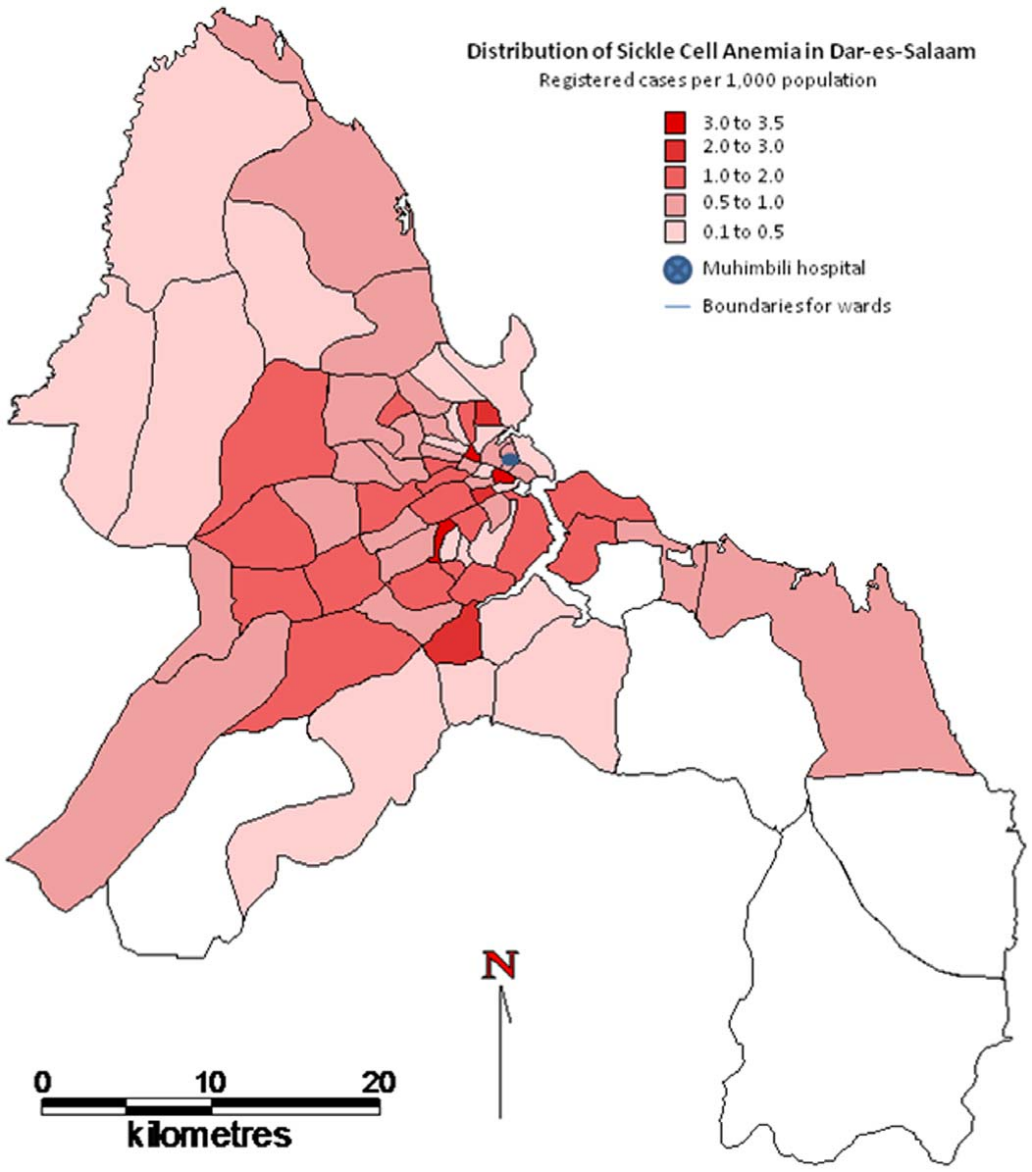
The geographical distribution of registered SCA patients who live in Dar-es-Salaam.

From March 2004 to March 2009, 1,725 (90.6%) people with SCA were enrolled, from 1,903 identified. The geographical distribution of registered SCA patients who live in Dar-es-Salaam is shown in figure 1, with 3.5/1000 as the highest population prevalence of registered cases.

### Age at enrollment and duration of follow-up (Table 1)

**Table 1:** Age at enrollment, duration of follow-up and age at death.

	Mean (SD)	Median	IQR	Range
**Age at entry (years)**				
Male (n = 856)	8.8 (7.4)	7	9–15	0–41
Female (n = 869)	10.5 (8.5)	9	7–12	0–48
**Duration of follow up (years)**				
Total (n = 1,564)	2.9 (1.7)	2.9	1.1–4.6	1 month to 5
**Age at death (years) (n = 86)**				
Male (n = 44)	12.7 (8.9)	11	4.5–18	2–37
Female (n = 42)	11.8 (8.9)	12	4–16	1–43

The median age at enrollment was 8 years [inter quartile range (IQR) 4–13] years, with the age distribution as follows: 169 (9.8%) <2 years; 365 (21.2%) 2–4 years; 477 (27.7%) 5–9 years; 519 (30.1%) 10–19 years and 195 (11.3%) >20 years. There were 856 (49.6%) males, who were significantly younger than females [Odds Ratio OR = 0.97 (0.96, 0.99); p-value <0.001]. Longitudinal information was available for 1,564 (90.6%) patients with 4,482 PYO, as 161 patients had only one visit. The median follow-up period was 2.9 years (IQR 1.1–4.6 years). Information on vital status (alive or dead) was not available on 209 (12.1%) patients who were lost to follow-up.

#### Mortality rate, age and place of death

Death occurred in 86 (5.7%) out of 1,516 patients; with only 20 (23.3%) occurring in MNH. The overall mortality rate was 1.9 (95%CI 1.5, 2.9) per 100 PYO. Since only 2 deaths were recorded in 46 children who were less than 2 years old at time of death or exit, the rate was calculated in three age groups, with the highest rate in those below 5 years old (table 2). The corresponding standardized mortality ratio was; 2.9 in children less than 5-years: 5.4 in children between 5–19 years and 1.1 in individuals 20 years and above.

**Table 2:** Mortality rates stratified by age at censoring or death.

Age (group)	Number of patients	Observation (yrs)	Number of Deaths	Mortality rate per 100 PYO (95%CI)
<5 years	243	303.0	22	7.3 (4.8–11.0)
5–19 years	1,053	3,325.6	47	1.4 (1.1–1.9)
>20 years	267	849.4	15	1.8 (1.1–2.9)
**Total**	**1,564**	**4,482**	**85**	**1.9 (1.5–2.9)**

Mortality rates for 1,564 SCA patients enrolled and with more than one clinic visit. The rates take into account date of birth and date of recruitment into the study.

#### Factors at enrollment associated with death and cause of death in hospitalized SCA patients

**Figure 2:**
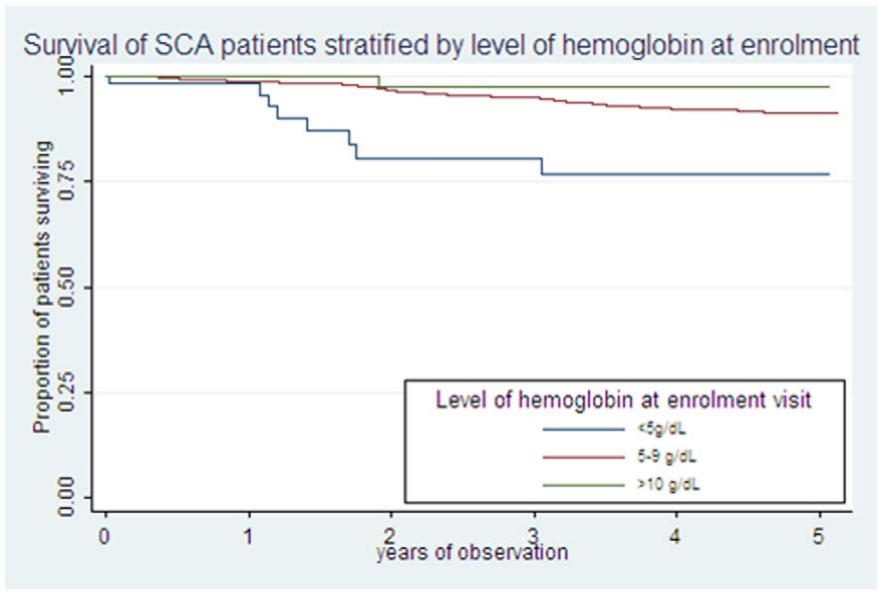
Survival of SCA during the study, stratified by level of hemoglobin at enrollment.

Table 2 shows the association between death and laboratory characteristics at enrollment of 1,516 patients. On univariable analysis, the SCA individuals who died had significantly lower hemoglobin and higher total and conjugated bilirubin (Table 3). Figure 2 shows the probability of survival depending on level of hemoglobin at enrollment. On multivariable analysis, hemoglobin [0.71 (0.59–0.84); p<0.001] and total bilirubin were independently associated with mortality [1.00 (1.00–1.01); p = 0.020]. Using categorical values, severe anemia, hemoglobin <5 g/dL [3.8 (1.8–8.2); p = 0.001] and high total bilirubin, ≥102 µmol/L [1.7 (1.0–2.9); p = 0.044] were significantly associated with mortality.

**Table 3:** Laboratory features at enrollment visit associated with survival in SCA patients.

Clinical features	Survived(n = 1,430; 94.3%)		Died(n = 86; 5.7%)		Odds Ratio(95% CI)	p
	n	n (%) or mean (SD)	n	n (%) or mean (SD)		
Age at enrollment (years)	1,430	9.5 (7.8)	86	10.2 (8.6)	1.01 (0.98–1.04)	0.408
White blood cell count (x10^9^/L)	1,307	15.9 (6.9)	78	15.7 (7.3)	0.99 (0.96–1.03)	0.849
Hemoglobin (g/dL)	1,306	7.6 (1.3)	79	6.9 (1.6)	0.73 (0.62–0.86)	<0.001
Mean Corpuscular volume (fL)	1,298	79.2 (9.5)	77	79.5 (10.9)	1.00 (0.98–1.03)	0.752
Reticulocyte (% of RBC)	832	12.6 (7.1)	33	13.1 (8.6)	1.01 (0.96–1.06)	0.670
Hemoglobin F (%)	1,297	6.3 (4.7)	63	6.6 (4.9)	1.02 (0.96–1.07)	0.575
Total Bilirubin (µmol/L)	1,263	69.7 (56.7)	79	87.5 (81.4)	1.00 (1.00–1.01)	0.010
Direct Bilirubin (µmol/L)	1,157	20.9 (34.2)	72	33.1 (57.6)	1.00 (1.00–1.01)	0.019
Indirect Bilirubin (µmol/L)	1,140	51.5 (53.6)	71	57.1 (60.4)	1.01 (0.99–1.01)	0.396
Lactate dehydrogenase (IU/L)	559	965.4 (483.7)	27	1,103.7 (339.3)	1.00 (0.99–1.00)	0.143
Aspartate transaminase (IU/L)	1,307	50.1 (28.4)	81	49.3 (24.1)	0.99 (0.99–1.01)	0.802
Alkaline phosphatase (IU/L)	1,310	265.6 (124.2)	81	276.7 (152.8)	1.00 (0.99–1.00)	0.442

Association between mortality and laboratory measurements at enrollment was explored using logistic regression.

## Discussion

This is the first study that presents rates and risk factors for mortality in SCA in Africa, the continent with the greatest burden of disease. The mortality in this cohort was 1.9 per 100 PYO which is similar to 3 per 100 PYO reported from the USA before use of penicillin prophylaxis[13]–[14], but is an order of magnitude higher than the current incidence of 0.13 per 100 PYO reported from the USA[4], [6]. This rate is likely to underestimate the mortality in most African settings, where resources such as skilled health care workers, investigations and treatment options are limited, since this study was conducted at a university teaching hospital, in an urban environment, where SCA individuals have a relatively higher socio-economic status with good access to health care.

The highest incidence of death in this study was in the first 5 years of life. This is similar to reports from Jamaica and USA in the 1980' and 1990s, where the highest rate was reported between 1–3 years[3], [15]–[17]. Evidence from previous research suggest that infection is the most likely cause of death in this period, with the proportion of deaths from infection reported to be 50% in the USA[3], [16], 28% in Jamaica[15], [17] and 20% in Dallas[6]. The prevention of pneumococcal infection with penicillin and the introduction of pneumococcal conjugate vaccine has been shown to be effective in reducing mortality[18]–[19] with improved survival rates of 84% in Jamaica[17], 86% by 18 years in Dallas[6] and 99% in London[7]. A recent review reported 42% reduction in mortality in SCA in USA, 0 to 3 years old, between two eras, 1995–1998 and 1999–2002[20]. There is compelling justification for implementation of these interventions in Africa to prevent deaths due to infections[8], [21]. This study highlights the high mortality and limited information on natural history of SCA in first 5 years of life. There is a dearth of information from children in early childhood as 2 deaths were recorded in 42 children in this age group and only 10% of this cohort was enrolled when less than 2 years old. This could be the result of children not coming to hospital because they are not symptomatic but since, the mortality was highest in the under 5-years age group, it is highly likely that many deaths occur in early childhood before a SCA diagnosis is made. This supports the importance of early identification of SCA children. Ideally, this should be at birth by NBS, and pilot studies have shown that NBS is feasible in Africa[22]–[24]. Thereafter, comprehensive care can be provided, with active interventions to prevent infections in early childhood, which is the most likely cause of death in this vulnerable period[25], [26].

Low hemoglobin at enrollment was associated with mortality as has been previously reported elsewhere[4], [7], [17], [27], [28]. There are limited options for interventions, with blood transfusion and Hydroxyurea being the two recommended therapies. Blood transfusion has been found to be beneficial in SCA with stroke, acute chest syndrome and peri-operatively in the West[29]. In Africa, it is mainly used for treatment of acute anemia in SCA[29]–[30], since there are considerable limitations of blood transfusion in Africa such as inadequate blood supply, risk of transmission of infections and alloimmunization. There is limited evidence to guide rational use of blood in Africa as well as studies to assess its impact on mortality. The alternative therapy is Hydroxyurea which increases hemoglobin and HbF levels, reduces hemolysis and blood transfusion requirement and has been effective in reducing mortality[31]. The association between HbF and death in our cohort was not significant, which may be due to a ‘healthy survivor effect’; with the cohort consisting of patients with high HbF and therefore being protected from mortality while those with low HbF have succumbed to early mortality. Better understanding of the burden and pathophysiology of anemia in SCA and the role of HbF in SCA in this setting could guide interventions.

There is increasing evidence of the role of hemolysis in pathogenesis of severe clinical complications in SCA, such as pulmonary hypertension and stroke[5], [32]. In this study we did not find an association between hemolysis and death. Although total bilirubin was an independent predictor, there was no association with other hemolytic markers such as unconjugated bilirubin, lactate dehydrogenase, aspartate transaminase and reticulocyte count. The lack of an association between hemolysis and mortality in this setting may be because hemolysis and the hyperhemolysis phenotype are not common in Africa[33] or those with this phenotype died before recruitment into this cohort. Furthermore, most of the studies that have shown this association were in adults[5]; the most recent study by Taylor *et al*, examined SCA patients over 30 years[32]. Since only 11% of our study population was above 20 years at enrollment, further studies are needed to examine the role of hemolysis, using direct measurement of free hemoglobin and comparison of markers during steady-state and acute episodes.

This cohort highlights key areas to improve survival in SCA and has identified gaps to guide further research. Over 85% of patients enrolled into our clinic-based cohort survived, suggesting that SCA patients in Africa are surviving beyond childhood. However, SCA patients have a high mortality based on mortality and median survival from this study. The estimated birth prevalence of SCA in Tanzania is 7/1000[1], [34]. If one assumes that this is representative of the urban population of Dar-es-Salaam, the population prevalence of the SCA patients attending the hospital is a maximum of 3.5/1,000. This leaves 50% of the SCA population unaccounted for and it is not known to what extent this reflects relatively well individuals not attending hospital, as opposed to loss due to premature death. The median survival in SCA patients in this cohort, comprising mainly older children and adults, was 33 years; which is 19 years less than life expectancy at birth (52 years) in Tanzania[35] and is also markedly lower than for SCA patients (40–60 years) in the USA[4] and Jamaica[9]. This median survival of SCA is most likely an overestimate, as it does not capture the individuals who died before diagnosis, or those who were lost to follow up. Modell *et al,* estimated that 6 million Africans would be living with SCA if average survival of affected children reaches half the African norm[36]. The burden of disease to individuals, communities and health systems has not been quantified, but these patients will suffer from anemia, painful crises, infections, stroke and other complications[2] and will require life-long care. It is also of note that 12% of those enrolled into the cohort were lost to follow-up and only 23% of deaths occurred in the hospital. Attendance may be improved with health education to SCA patients and their caregivers on the importance of regular follow-up and comprehensive care. This finding also highlights the need to develop clear guidelines of health services for SCA at home and within health facilities in the community. We identified risk factors for mortality, which should allow targeting of interventions to high risk patients. Although most studies use steady-state laboratory values (average well-visit routine measurements)[3], [4] in this study we used factors at enrollment visit, as SCA patients in Africa may be seen at a health facility only once and most deaths occurred outside MNH. Finally, although the mortality was highest in the young children, this is most likely an underestimate as many children are likely to have died before they could be referred to the hospital clinic or diagnosed with SCA.

This study provides a description of mortality rates in SCA amongst a hospital-based cohort in Dar-es-Salaam, Tanzania. As expected, there is a high mortality in SCA, with children significantly more affected. Although we have provided data that will guide initial policies, the study has highlighted the areas for further research. We propose that priority should be given to the establishment of NBS to detect SCA patients at birth and allow active prevention of infection. A birth cohort will also provide accurate survival data and information on causes of morbidity and mortality in early childhood. Furthermore, since anemia was associated with mortality, detailed studies to identify causes and clinical trials to determine appropriate interventions to ameliorate anemia are needed.
